# Ovariotomy

**Published:** 1890-07-19

**Authors:** 


### OVARIOTOMY.

Mr. Meredith in his recent address on the present position of abdominal surgery, delivered before the London Medical Society, remarks that the present range of abdominal work includes operative measures for diseases of every organ or structure contained within the abdominal cavity, including the liver and gall-bladder; the spleen and the pancreas; the kidneys, ureters and bladder; the stomach and intestinal tract; the uterus and its appendages; and finally the peritoneum itself, as well as the tissues underlying its various folds. Only a few years ago abdominal surgery was practically limited to the removal of ovarian tumours; and Mr. Meredith might have added that thirty years ago, an eminent surgeon stated that he could not conceive any operation for the removal of an ovarian tumour justifiable. Among the improved methods of treatment of ovariotomy cases, Mr. Meredith mentions the abandonment of the clamp in favour of the intra-peritoneal ligature, and he says "the use of the clamp was undoubtedly the most direct factor in the causation of mortality." The next most important advance was the application of Lister's antiseptic system. It may, however, be remarked that Mr. Meredith seems to think perfect cleanliness is of as much importance as antiseptics. For he writes, "Without entering into the vexed question of the relative value of antiseptics and of plain water, he will merely express his conviction that the teachings of Lister, by directing attention to the advantages of cleanliness, in the widest acceptation of the term, have done more to promote the present position of abdominal surgery than anything else." Drainage by improved tubes has done much to render drainage safe and efficacious. The plan of flushing out the peritoneal cavity has proved of immense value. But the addition of antiseptic substances, whether carbolic acid, corrosive sublimate, or what not, is fraught with risk of poisoning, and can do no possible good." The routine administration of opium as formerly recommended is a mistake, owing to the restraining influence exerted by the drug on the processes of absorption and excretion. As regards saline purgatives for the treatment of early symptoms of peritoneal mischief, Mr. Meredith can only conceive benefit from them where no actual obstructive interference with intestinal peristalsis is present. If obstruction, whether due to fæcal impaction or to adhesive inflammation, is present, attempt at purgation, by inducing paresis of the bowel above the seat of difficulty cannot but result in harm. In cases of this nature, Mr. Meredith prefers to administer belladonna in 20 minim doses of the tincture, which, by inducing peristalysis will usually overcome the difficulty.

On referring to other authors we find Mr. Jacobson stating, in agreement with Mr. Meredith, that the intra-peritoneal method is now most universally adopted. Mr. Thornton also bears similar testimony. As regards antiseptics Mr. Jacobson does not even mention them. But after applying the ligature and stopping all bleeding, Mr. Jacobson says the next step is the so-called "toilet," which means most thoroughly sponging out the pelvis by introducing again and again warm sponges, until they return dry and colourless. Mr. Jacobson also says that the question of drainage must depend mainly on the completeness of the "toilet" of the peritoneum, the probability of any subsequent oozing, and the possibility of any septic fluid having entered the peritoneal cavity. He is in accord with Mr. Meredith in recommending a glass tube. Mr. Jacobson also advises less routine in the matter of morphia, and he endorses Mr. Thornton's recommendation of an enemata, some few hours after the operation, of beef tea. In cases of threatening or actual peritonitis, when only flatulence and some distensions are present, they may yield to the passage of a long rectal tube, and the injection of a pint of water, with or without turpentine. If vomiting and tympanitis continue, opening the wound and irrigation of the peritoneal cavity with a 2 per cent. of warm boracic lotion should be tried.

The accidents during ovariotomy are thus enumerated: *fainting*, which should be treated in the usual manner; *vomiting*, which demands expedition, and pressure of the abdominal wall against the viscera by an assistant; *separation of the parietal peritoneum*, which is avoided by care in the operation; *rupture of the cyst*, which demands additional care in the "toilet"; *injury to the viscera*, the bladder, small intestine, rectum, and ureter, being most likely to suffer, which, if occurring, may require different treatment. In the case of injury to the bladder it becomes a question whether the operation should be deferred or completed, and there are no rules on which to base an opinion. In the case of injury to the intestine, sutures must be used. If the ureter is injured, it will be wisest to ligature the lower end, and, if possible, bring the upper end out of the wound. In one case of the kind it was found necessary to perform nephrictomy afterwards. Lastly, instruments, sponge, or forceps have been left in the abdomen. The fact that this accident has occurred with operators of large experience demonstrates the necessity of care, counting instruments and sponges carefully afterwards, and allowing no tearing of sponges.

